# The challenges in managing the growth of indigenous children in Perak State, Malaysia: A qualitative study

**DOI:** 10.1371/journal.pone.0265917

**Published:** 2022-03-23

**Authors:** Chii-Chii Chew, Xin-Jie Lim, Lee-Lan Low, Kin-Mun Lau, Maziana Kari, Ummi Kalthom Shamsudin, Philip Rajan

**Affiliations:** 1 Clinical Research Centre, Hospital Raja Permaisuri Bainun, Ministry of Health, Ipoh, Malaysia; 2 Institute for Health Systems Research, Ministry of Health, Ipoh, Malaysia; 3 Perak State Health Department, Ministry of Health, Ipoh, Ipoh, Malaysia; UCSI University, MALAYSIA

## Abstract

Indigenous peoples in Peninsular Malaysia, known as *Orang Asli*, have been associated with the problem of malnutrition. Approximately 40% of their children are underweight. Indigenous peoples’ distinct social, cultural, and economic traits, which differ from those of the dominant communities in which they live, may pose significant challenges for health care providers (HCPs) in addressing the malnutrition issue. This study explores challenges encountered by HCPs, with at least six months of experience in monitoring the growth parameters of *Orang Asli* children residing in Perak State in Peninsular Malaysia. A cross-sectional study was conducted between December 2020 and June 2021, involving three focus group discussions and three in-depth interviews. Thematic analysis was used. A total of 19 participants (6 nurses, 5 nursing managers, 4 medical officers, 2 nutritionists, a family medicine specialist, and a paediatrician) took part in this study. The challenges were summarized into four themes: (I) accessibility to nutrition, (II) accessibility to healthcare services, (II) skills of HCPs, and (IV) challenges of implementing nutrition programs. The inability of the *Orang Asli* children to access nutritious food was due to poverty, different perceptions of life priorities, and the practice of food taboos among the communities. Inadequate infrastructure and transportation discourage parents from bringing their children to healthcare facilities. The belief in and preference for traditional healing, the practice of semi-nomadic lifestyles, and fear of HCPs and their timid nature were factors that prevented *Orang Asli* children from accessing healthcare services. HCPs need to equip themselves with cross-cultural communication and interaction skills and adapt their skills to environmental challenges to overcome unexpected encounters in mobile clinics. The non-exposed food items, the risk of food basket sharing with other family members, and community feeding programs’ coordination were the challenges to be addressed when implementing nutrition programmes for *Orang Asli* children. The challenges of HCPs are multifactorial and require a multifaceted approach. There is a need for joint efforts of stakeholders, from communities and non-governmental organisations (NGOs) to the health authorities, to address the challenges of HCPs.

## Introduction

Approximately 370 million self-identified indigenous peoples reside in nearly 90 countries worldwide. The indigenous peoples are vulnerable, marginalized, and underprivileged populations [1]. Culture, social networks, history, racism, socioeconomic disadvantage, psychological distress, strong social ties to family and kin, and mistrust of non-indigenous people are all factors that influence indigenous people’s health and behavior [2–4]. These factors are complex, and dealing with the health of indigenous peoples remains a challenge for health professionals and health organizations.

One of the challenges encountered was indigenous children’s undernutrition, especially those under 5 years of age, which is prevalent in developing countries [5]. In Brazil, about 6% and 26% of indigenous children were underweight or stunted, respectively [6]. In Africa, one of the semi-nomadic ethnic groups known as the Maasai has nearly 60% of their children under the age of five who are physically stunted, suggesting chronic malnutrition [7]. Childhood malnutrition raises mortality rates and disease burden [8]. Indigenous peoples in Latin America have greater mortality and morbidity rates than the rest of the population [9]. A similar trend was found in Brazil, where indigenous groups had significantly higher infant mortality rates than the general population [10].

As with indigenous people in other countries [11–14], the indigenous people in Peninsular Malaysia, known as *Orang Asli* [15], are not exempted from the problem of childhood malnutrition, bearing severe consequences for not just the individual, but also the health system [16–18]. Children under the age of five are at risk of malnutrition and are often categorized as underweight [16, 19–21]. A range of 39% to 64% of under-five *Orang Asli* children in Pahang and Selangor State in Peninsular Malaysia were found underweight [16, 22, 23]. Perak state, located northwest of Peninsular Malaysia, recorded the second-highest number of *Orang Asli* (53,299, 29.9%) after Pahang State (67,506, 37.9%) [24], and had approximately 33% of *Orang Asli* children under the age of five were underweight [25], and nearly 40% of *Orang Asli* children under the age of two were underweight [26].

Globally, most indigenous peoples are at a socio-economic and political disadvantage. The socio-economical limitations of the indigenous community are often associated with poverty, and this has been a significant risk factor for underweight [27]. Geographically, the majority of indigenous people live in rural areas or locations, preventing them from accessing services and facilities, resulting in a lack of access to clean drinking water, toilet facilities, housing, and cooking fuel. Poverty and disadvantageous environmental factors predispose indigenous children to underweight [21, 28, 29]. Similar to the phenomenon of indigenous people in other countries, one local study reported three factors associated with underweight among *Orang Asli* children: poverty, no or low-level maternal education, and maternal unemployment [19].

Various measures have been taken by governments of different countries to tackle indigenous children’s undernutrition [2, 4, 30–33]. The challenge was that the conventional health service model may not be administratively fit for all indigenous peoples [34], and some of the health services are commonly resisted by some of the indigenous population [35]. A distinctly different need for health services that are appropriate, acceptable, affordable, accessible, and participatory has been expressed by some indigenous peoples [36]. The Ministry of Health in Malaysia has been taking multiple approaches to improve the problem of malnutrition among *Orang Asli* children. Healthcare professionals in primary care, including general practitioners, community-based nurses, and nutritionists, have been identified as stakeholders that can play a meaningful role in malnutrition identification and management in the community [37]. Besides static healthcare facilities, Malaysia also has mobile clinics that provide primary health care to indigenous people living in remote areas, as well as transit centres where patients and their accompanying guardians are housed while waiting to be transferred to a hospital for treatment or follow-up. Meals were provided for the patient and two accompanying family members [38].

The nutritional programs, better known as the ‘Rehabilitation Program for Malnourished Children’, introduced by the Ministry of Health Malaysia, aim to help severe and moderately underweight children, below national poverty line, aged between 6 months to 6 years, recover from malnutrition. The help was given to the children in the form of food baskets on a monthly basis [39]. The *Orang Asli* population, with nearly 77% of them living in poverty, were recruited into this programme too. Besides the food basket, the "Community Feeding Programme", comprising ready-to-use therapeutic food (RUTF) and supplemental feeding, is being introduced to the *Orang Asli* children. RUTF is a paste containing energy-dense and enriched micronutrients for therapeutic nutrition, while supplemental feeding is given five times per week and consists of a glass of milk, biscuits or cereals, and multivitamins containing iron and fish oil. The “Community Feeding Programme” also consists of community empowerment activities that include enhancing knowledge of personal hygiene and nutrition via health screenings, cooking classes, and healthy food preparation [40].

Despite all the efforts by the Ministry of Health Malaysia, the 2020 Global Nutrition Report found that Malaysia made little progress in meeting the targets for undernutrition among under-five non-indigenous children (stunting 21.8% vs. wasting 9.4% and being underweight 14.1%) [41, 42] and indigenous groups, especially the *Orang Asli* children (stunting 45.8%, wasting 42.3% and being underweight 59.1%) [43]. While the problem of underweight among *Orang Asli* children has been prevalent [19, 23, 26], the issues associated with this problem, apart from the inherent risk factor of *Orang Asli* populations, have not been investigated explicitly.

A study shows that healthcare personnel in the indigenous primary healthcare service are crucial in building a stable workforce to meet the health needs of indigenous people [32]. They are required to be equipped with good skills, motivated and have adequate experience to sustain effective healthcare delivery to the indigenous people [32, 44]. Healthcare providers, being the key players in growth management, would thus provide valuable insight into the issues surrounding the management of growth in *Orang Asli* children. The perspectives of healthcare providers with regard to these issues, including the challenges they encounter, are not explored. To the best of researchers’ knowledge, there is no research reporting these findings. The findings of the study could be used to improve current health services for *Orang Asli* children. This study aims to explore the challenges of healthcare professionals managing the growth of *Orang Asli* children.

## Methods

### Study design

This qualitative study explores the reality experienced by healthcare professionals (HCPs) in managing the growth of *Orang Asli* children. This study was conducted between December 2020 and June 2021 using inductive research approaches.

### Study setting & sampling

Purposive sampling was employed to get diverse HCPs with different experiences of handling *Orang Asli* children from different localities (remote and fringes) for at least 6 months in the districts of Perak State. The districts selected were *Hulu Perak*, and *Kinta* districts, mainly attributed to their vastly different geographical locations between these two districts. *Hulu Perak* is located in the north, with most of the townships in this district being considerably sub-urban, whereas *Kinta* is located in the centre of Perak State. It is where the State Capital (Ipoh City) is located, and is considered an urban area, although most of the *Orang Asli* villages are located in peri-urban areas or town fringes. This implied that the population of *Orang Asli* who resided in these districts could have been practising a distinctively different socio-economic status and lifestyle. For instance, a study reported that the economic activity of *Orang Asli* (*Temiar* tribe) community in *Hulu Perak* has been actively engaged in forest product gathering [45]. On the other hand, the *Orang Asli* population in the central part of Perak, including Kinta district, is mainly comprised of the *Semai* tribe. The *Semai* tribe is divided into the highland and lowland *Semai*. The highland one is more suited to activities including hunting, fishing, gathering, and swidden agriculture, while the lowland have traditionally participated in the labour force and sought work in small-scale jungle produce commerce and are now more exposed to the modern economy [15]. Furthermore, a previous study suggested that *Orang Asli* lifestyles were different between those who lived nearer to the town and those living on the fringes of town as compared to those living in the interior areas [24]. In addition, *Hulu Perak* was identified as one of the districts that recorded the highest number of children with malnutrition [46]. Selecting HCPs to manage the health of *Orang Asli* populations who were located in two extremely different situations allows researchers to explore HCP experiences in different contexts based on the settings of *Orang Asli* populations. Paediatricians, family medicine specialists, medical officers, staff nurses, nutritionists, and nursing managers were recruited for this study since they are extensively involved in providing integrated management of growth in the *Orang Asli* population. This setting and healthcare structure are applicable to other states in Peninsular Malaysia where the *Orang Asli* are populated [47, 48].

### Data collection

The HCPs were selected from a personnel list obtained from the Perak State Health Department. They were first invited through telephone calls, and those who agreed to participate were grouped according to their professions for focus group discussions (FGD). The implementation of the Full Movement Control Order (FMCO) by the Malaysian government from June 1st to 28th, 2021 in response to the COVID-19 pandemic has limited the physical attendance of the participants. Researchers decided to conduct virtual modes for data collected within the period of FMCO, while FGD conducted before FMCO was done via face-to-face mode. Two FGDs were conducted face-to-face. Seven potential respondents, consisting of medical officers and family medicine specialists, were invited. Five respondents participated, while two medical officers declined the invitation due to a busy schedule and maternity leave, while the other group recruited six nurses who have been working in the mobile clinics. The face-to-face FGD was conducted in a meeting room of a research centre and not at the intuitions where the HCPs are currently working; the room was deemed sufficiently private for the researchers and participants, and no other person was allowed to be present during the discussion. Meanwhile, a virtual FGD was carried out among five nursing managers using Google Meet®. For the FGD invitation, there is no declination for staff nurses or nursing managers.

Two female researchers (CCC and XJL) were moderators for FGD who had no prior knowledge of the participants in person prior to the discussion. The researchers are HCPs with two years of experience in qualitative research, and XJL has been a paediatric doctor for five years and is currently a candidate for a master’s program in public health, while CCC is a pharmacist by profession and holds a master’s degree in social and administrative pharmacy. Both researchers are currently working in research with at least 5 years of experience in research.

In each FGD, participants were prompted with questions asking about their experiences and challenges in (i) managing the growth of *Orang Asli* children, (ii) running mobile clinics to reach out to *Orang Asli* in the remote areas for those who were involved in the mobile clinic, and (iii) their suggestions for overcoming the challenges and improvements, if any. The moderators facilitate the discussion by ensuring the discussion covers the areas of procedures, staffing, facilities, transportation, nutritional programs, and guidelines (growth chart and child health record book) needed for managing the growth of *Orang Asli* children. The guide developed for FGD was based on literature and discussed with a senior qualitative researcher (LLL).

Subsequently, the same researchers (CCC and XJL) were the interviewers for in-depth interviews (IDI). The IDI were employed when themes emerged, suggesting the researchers gauged the information from key informants—a paediatrician in a tertiary hospital and two nutritionists who had experience handling malnutrition of *Orang Asli* children. All three IDI were conducted virtually. The paediatrician was prompted with semi-structured questions with regards to the experience and challenges of managing malnourished *Orang Asli* children requiring admission to the tertiary hospital, while nutritionists were asked for their experience and challenges in implementing nutritional programmes, particularly on the topics of food baskets, community feeding programmes, implementation process, and training for nurses and medical doctors who were involved partly in the programs’ implementation. No interview was repeated and data saturation was reached after an additional three IDI.

The participants were briefed on the study, and they were given sufficient time to ask any study-related questions. Then, written or verbal informed consent, depending on the mode of data collection, was obtained from the participants before data collection. Participants’ demographic information was collected, and the data for FGD and IDI were audio recorded. Approximately 3 hours and 1 hour were taken for FGD and IDI, respectively. No observation notes or non-verbal communication were collected as the researchers did not plan to analyse these data.

### Data analysis

All the audio recordings were transcribed verbatim by two researchers at the end of each session. The transcriptions were not returned to the participants for comments. However, the debriefing of discussion for FGD and IDI was conducted at the end of each session to reach consensus and ensure researchers understood the information captured. The quotes identified from discussions that were conducted in the Malay language were translated into the English language by a bilingual researcher and then cross-checked by another bilingual researcher for accuracy. The data was managed using a Microsoft® 365 Excel spreadsheet, and thematic analysis was performed. CCC and XJL were involved in developing codebooks and conducting open coding independently at the end of each FGD and IDI. Inductive analysis was performed for the initial coding based on the research objectives. The codes generated separately by the two researchers were then discussed, compared, and modified. The codes were collated to generate descriptive preliminary themes and sub-themes. Then, the two researchers engaged a senior qualitative researcher (LLL) for a series of discussions and feedback to fine-tune the themes and sub-themes. Constant comparisons were made to the themes generated by the data to confirm the endpoint of data saturation. The endpoint was considered achieved once sufficient data had been collected to account for emerging themes [49, 50]. A consensus on the themes and subthemes generated was reached among the researchers. The findings were not provided to the participants for feedback; however, we had a series of discussions and presentations of the findings among the peers and stakeholders during the analysis process.

### Ethics approval and consent to participate

Ethical approval was obtained from the Malaysian Medical Research Ethical Committee (MREC) via registration with the National Medical Research Registry, Ministry of Health Malaysia under protocol registration number of NMRR-20-2454-56947 (IIR). This study was conducted in accordance with the Malaysian NIH Guidelines for Conducting Research in MOH Institutions and Facilities. Written informed consents were obtained from participants for the face-to-face discussion, while verbal informed consents were taken from the participants in the virtual discussion, as approved by the MREC.

## Results

### Characteristics of participants

A total of 19 participants participated in this study. The participants ranged in age from 28 to 54 years old, with the majority being female (77.8%). They had worked for the Ministry of Health for three to 30 years, and they had been involved in the care of *Orang Asli* children for at least six months to 17 years (Table 1).

**Table 1:** Demographic characteristics of the participants.

Characteristic		n = 19, n (%)
Age, median (IQR)		40.0 (9.0)
Year of experience working in the MOH, median (IQR)		16.0 (9.0)
Year of experience managing the growth of *Orang Asli* children, median (IQR)		4.0 (10.0)
Gender	Male	4 (21.1)
	Female	15 (78.9)
Profession	Nurse	6 (31.6)
	Nursing Manager	5 (26.3)
	Medical Officer	4 (21.1)
	Nutritionist	2 (10.5)
	Family Medicine Specialist	1 (5.3)
	Paediatrician	1 (5.3)

MOH: Ministry of Health Malaysia

### The challenges of health personnel in managing the growth of *Orang Asli* children

The challenges of HCPs in managing the growth of *Orang Asli* children were considered with regard to multiple dimensions. Firstly, the *Orang Asli* communities’ cultural and social-economic background result in *Orang Asli* children’s inability to access sustainable resources for childhood nutrition and healthcare services, the key elements for growth. Secondly, the cultural challenges experienced by HCPs emphasized the need for cross-culturally competent HCPs to tackle issues associated with cultural differences between non-indigenous HCPs and *Orang Asli*. Various unexpected environmental challenges in delivering mobile healthcare services highlighted the need for adaptive skills to be included in the in-service nutrition training for HCPs. Thirdly, the implementation of nutritional programs, an important component of *Orang Asli* children’s growth management, was accompanied by a number of challenges throughout implementation. The themes and subthemes are summarised in Table 2.

**Table 2:** Themes and subthemes generated.

Themes	Subthemes
Accessibility to childhood nutrition	• Poverty • Perception of life priorities • Practice of food taboos.
Accessibility to healthcare services	• Inadequate infrastructure • Financial constraint (transportation and meals expenditure) • Belief and preference in traditional healing • Semi-nomadic lifestyles • Fear of healthcare providers
Skills of healthcare providers	• Cross-cultural communication and interaction • Adaptive skills to environmental challenges
Challenges in implementation of nutritional programs	• Cultural appropriation of food. • Unintended use • Food Basket Sharing • Community feeding programs

#### Accessibility to childhood nutrition

‴Accessibility" is described as being able to reach or obtain something easily [51]. *Orang Asli* children’s undernutrition was a result of the inaccessibility of diverse, nutritious foods where the communities have been living with barely sufficient resources.

*Unfortunately, at the age of one year, the (Orang Asli) children were good at eating sweet potatoes. We (the staff nurses) said, "That’s not allowed." How can we say it’s not allowed when that’s all there is in their village (FGD, nurse).*

As described in the sub-themes below, poverty, different perceptions of life priorities, and the practice of food taboos exacerbated the situation.

*Poverty*. According to the HCPs in this study, *Orang Asli*’s access to food was hampered by poverty, which occurs when parents do not have adequate money to purchase nutritious food for their children. Poverty makes *Orang Asli* children vulnerable to food insecurity. It drives them to eat more calorie-dense, nutrient-deficient foods, which may predispose them to the risk of poor growth and development.

*I believe poverty is the underlying cause of malnutrition; parents’ lack of consistent income led to inadequate nutritious food supplies. They don’t have enough money to purchase food, so they must rely on natural resources like bamboo shoots and cassava leaves…children of Orang Asli parents who relied only on forest revenue are often malnourished. On the other hand, children whose parents worked in a factory or on an oil palm plantation thrived more steadily (FGD, medical doctor).*

*Perception of life priorities*. Perceptions of HCPs in this study indicated that personal needs were prioritised differently in the *Orang Asli* community. Some of the *Orang Asli* parents were deemed to have placed a greater priority on other aspects than food.

*They live in decent houses, but they don’t consider food an essential thing for them, so they may go out in the morning without it, their children can simply drink coffee at home for breakfast and fill their stomachs this way…when they have money, they seem unable to differentiate between what is important to them and what is not. From what I (medical doctor) can gather, they enjoyed spending money on entertainment, cosmetics, and gadgets…they were able to buy hair colouring products, and some have gone for hair rebonding, despite the fact that their children are starving (FGD, medical doctor).*

*Practice of food taboos*. HCPs noticed that food taboos persist in *Orang Asli* dietary customs and often restrict people from eating particular foods for a variety of reasons, including the avoidance of certain foods to prevent adverse effects on human health and the belief that some foods are dirty to consume. *Orang Asli* culture and belief in food intake greatly affect the accessibility of children to a variety of nutritious foods.

*They practice ‘pantang’ (taboo) in which children less than one year of age cannot take protein, anchovies were not allowed…they raise chickens in the village, but they don’t eat them because they feel the home bred chickens were dirty, which feed on the ground may be taking poop,…every time when we (the HCPs) suggested them for the other options of protein, for example, beans, they would always say the food can cause bloating (FGD, medical doctor).*

#### Accessibility to healthcare services

The inability of *Orang Asli* children to access healthcare services was not confined to the obstacles that *Orang Asli* parents experienced in sending their children to healthcare services, but also to the difficulties that HCPs faced while providing outreach services to *Orang Asli* children.

*For example, when we were going to the villages, we needed to cross the river (by wading), and the water level was up to our thighs, (so) we were all wet… (Can you) imagine the white uniform (that we wore) when we waded out to the villages? It (the uniform) then turned sticky (FGD, nurse).*

The factors that explain the bidirectional challenges include underdeveloped infrastructure, unaffordable family expenses during a child’s hospitalization, the practice of traditional healing and semi-nomadic lifestyles of certain *Orang Asli* communities, as well as the natural characteristics of *Orang Asli*, particularly fear and shyness when seeing outsiders such as HCPs, preventing their children from seeking healthcare services.

*Inadequate infrastructure*. *Orang Asli* often reside in remote areas with inadequate infrastructure and harsh terrain. In remote areas, the infrastructure may restrict the modes of transportation. Most of the settlements lack year-round road access, just rugged and difficult dirt tracks that can be traversed only by four-wheel drive vehicles. The rainy weather made driving more difficult. The paths were muddy and treacherous, and puddles of water were everywhere. For villages that were only accessible by water route, transportation to and from them was dependent on weather and seasonal circumstances, which restricted both indigenous people and HCPs from traveling safely.

*We were tired when we had to cross the river and hike uphill, and the vehicle sometimes got stuck in the forest. We may have to reschedule the trip owing to vehicle breakdowns, fallen trees and branches blocking the narrow pathways, or staff members taking sick leave, including boatmen and drivers…the rainy season has hindered mobile clinic operations, it is difficult for us to reach the villages. When it’s raining heavily, we can’t go by boat, we must seek shelter and wait for the rain to stop…we tried to avoid boat trips at night, as the boat may collide with falling branches on the water’s surface, this is much too risky for us (the HCPs)… however, there were occasions when we had to go by boat at night due to emergency events (FGD, nurse).*

The lack of infrastructure was reflected in the absence of appropriate buildings for HCPs to provide healthcare services in the village. In the event of unanticipated events, HCPs have to set up an outdoor mobile clinic. HCPs were vulnerable to the threat of stray animals under these conditions. The lack of a proper building for mobile clinic sessions led to inaccurate anthropometric measurements. This was attributed to uneven surfaces when the measurements were conducted outdoors.

*The designated room was locked by the Tok Batin (village leader) who brought along the key to the forest, then we (the HCPs) had to conduct the clinic sessions underneath the trees, and hence, sometimes we had to face threats like stray dogs…not all areas that we have our clinic session in a proper building or buildings provided by the government. Sometimes, we will use places like rented houses and that rented house would be made from bamboo. This would cause our weighing scale to give inaccurate readings as the uneven floors were made of bamboo (FGD, nurse).*

*Financial constraint (transportation and meals expenditure)*. The shortage of healthcare facilities and limited infrastructure in *Orang Asli* communities contributes to inadequate connectivity between communities and healthcare professionals. Despite healthcare services provided at government-funded facilities being free-of-charge to them, they may be required to spend significant transportation fees to reach the nearest healthcare facility. The condition was worsened by the practice of indigenous kinship. An *Orang Asli* child is admitted to the hospital. Not only is the sick person, but the whole family may need to accompany the child. Subsidies from the government are not available to cover the cost of travel and meals for the whole family. In addition, there may be an extra structural financial barrier for *Orang Asli* living in remote areas to accessing healthcare services in urban areas due to the incurred expenses while traveling to seek healthcare.

*Most of the parents were cooperative when hospitalisation was needed for their severely malnourished children. The financial problem was their main concern because the parents who brought the child to the hospital needed food too. They won’t tell us, but according to my medical assistant, if they went back home to pack their bags for a few hours and did not come back to the clinic, they actually went to borrow money. Otherwise, they will tap rubber and sell it instantly for cash (FGD, medical doctor).*

*Belief and preference in traditional healing*. The HCPs described how some of the *Orang Asli* communities took care of their health via traditional healing practices and sought treatment from traditional practitioners, which are often linked with spiritual beliefs. Traditional healers have a variety of roles, including employing counter sorcery to remove the evil influences causing illness. The disparity between the fundamental concepts of modern medicine and traditional indigenous health beliefs provides a barrier to *Orang Asli* children accessing healthcare services.

*When a person was sick, he or she was not allowed to leave the house. I had difficulties bringing the ill child to the hospital for treatment. I needed to hunt for traditional healers who could break the spell; after that, the child was only permitted to follow us to the hospital (FGD, nurse).*

*Semi-nomadic lifestyle*. Our HCPs noted that some *Orang Asli* continue to live a semi-nomadic lifestyle. They continue to migrate seasonally from one of their recently established permanent settlements to another. They were notoriously difficult to locate. As a result, it poses significant difficulties for HCPs in tracking children’s growth.

*They would disappear for months, perhaps moving to Kelantan (another Malaysian state) or Gerik (another district in Perak State), and then return to us (the HCPs)… once migrated, their growth was not closely monitored… the graphs shown on the growth charts were incomplete (FGD, nurse).*

*Fear of HCPs*. An observation shared by the HCPs during FGD was that the *Orang Asli* were distrustful and apprehensive of outsiders. This may have a detrimental effect on clinical outcomes, such as indigenous people’s adherence to clinic attendance and healthcare management.

*As our boat approached the village, everyone fled into the forest. We had to convince them to attend the clinic; otherwise, they would not…some mothers even send their husbands to tell us about their children’s health conditions; they refuse to bring their children to visit us, leaving us unable to assess them…they were fearful of unfamiliar HCPs, but if they get familiar with you, they will trust you (FGD, nurse).*

### Skills of HCPs

The skills of HCPs in dealing with the *Orang Asli* community require HCPs to be equipped with communication and adaptive skills that are cross-cultural and accepted by the *Orang Asli* community.

*If we went to their (Orang Asli) house and sat with them, they asked, "Misi (staff nurse) tak geli (not feeling disgusted)?" I answered: "Why should I feel geli (digusted)? I’m becoming an Orang Asli too, so I would sit with you”. We have to know their (Orang Asli) culture, what their practises are, and what food they eat. Why can’t they eat certain foods? What is their food taboo (that causing) they can’t eat (certain food)? (FGD, nurse)*

Cross-cultural competence refers to the capability of HCPs to demonstrate cross-cultural communication and interaction skills to perform effectively while embracing the cultural diversity and cultural differences of clients in order to meet their needs as well as attain a desired clinical outcome [52]. Moreover, acquiring adaptive skills, which are defined as practical, daily abilities necessary to work and meet environmental challenges [53], is essential for HCPs in mobile teams who will anticipate various unexpected encounters.

#### Cross-cultural communication and interaction

HCPs recognize the importance of cross-cultural communication when interacting with *Orang Asli*. They are more likely to have a positive and beneficial relationship with healthcare providers, which results in better health outcomes. Culturally sensitive communication is also essential for providers to earn the patient’s and family’s trust. Culturally sensitive communication may be achieved by showing respect for the patient’s and family’s culture by paying attention to *Orang Asli*’s values, beliefs, and practices. A scenario shared by the nurses was as follows:-

*Patients who are supposed to be admitted to transit (temporary shelter for Orang Asli patients to receive basic treatment while awaiting transfer to hospital) but the patient refuses. They said they see ghosts in transit and they need a spell in transit…luckily our nurses were quite good at talking to the patients, their family members, and even the ‘Tok Batin’ (Head of Orang Asli Village)…so we managed to persuade them to admit to the transit, (we) just needed to know how to tackle them (FGD, nurse).*

#### Adaptive skills to environmental challenges

In-service nutrition training has been restructured such that it prepares the HCPs for real-life challenges when they enter *Orang Asli* villages. The HCPs would then be able to deliver healthcare services on sites adapted to structural and environmental challenges.

*We changed the way we organise (nutrition management) courses in order to attract HCPs by giving them the concept that if they don’t see these cases in the real (conventional) clinic, they will see them in the mobile team. Some of the nurses not used to working in a mobile team were not sure how to find the right places to run anthropometric measurements inside the village… So, we do teach them in this course to give them an actual idea (IDI, nutritionist).*

### Challenges in the implementation of nutritional programs

There have been some barriers in the mechanism of HCPs implementing nutrition programmes, in the form of food baskets and community feeding, to the Orang Asli community in order to promote the growth of their children.

*Let’s say the patients (Orang Asli children) move to some other places, we have to contact the next place where they will go. Let’s say they moved to Kelantan (a state adjacent to Perak state in Peninsular Malaysia), I would have to contact Kelantan (mobile team of Orang Asli in Kelantan State) … and inform them the patients are going there. So they must continue to receive food baskets from there, but it is not always easy to know where they go because they (the Orang Asli) do not always tell us where they want to go. They just mentioned ‘masuk hutan’ (going into the jungle) or something like that, so which ‘hutan’ (jungle) they go to we don’t know (FGD, nurse)*

Several challenges were associated with supplying food baskets, including cultural appropriation of food basket content for the food culture of *Orang Asli*. Nevertheless, some of the food basket limitations can be improved by the introduction of a community feeding program.

#### Cultural appropriation of food

The food items in the food basket may not be culturally appropriate due to dietary taboos, food preferences, and food preparation methods and skills of the *Orang Asli* community. As a result, some parents may not be using the food basket to its fullest capacity.

*We sometimes provided full cream milk powder, but there were some taboos that prevented them from accepting it…based on my interactions with the Orang Asli community, they may not know how to prepare the food (based on their conventional practices). When I checked their food basket, they were not taking cornflakes because they did not know how to prepare cornflakes for their children (FGD, nurse).*

#### Unintended use

As observed by some of the HCPs, the food basket was associated with unintended consequences, and the malnourished child did not benefit. With external help, it reduces the need for *Orang Asli* parents to make their own living.

*I saw that in the village I visited, people would sometimes go rubber tapping, fishing, and planting vegetables in the forest, but once they received the food basket, they would stop working since they had received food aid. We should offer milk only, not baskets with a wide range of items…they did not return to work after getting aid, (and) therefore we must provide them in sequential portions. I once observed an Orang Asli who gathered food baskets including rice, frying oil, and anchovies and sold them. He used the money to stay at a hotel for one night (FGD, nurse).*

#### Food basket sharing

Despite the fact that the food basket was distributed to malnourished *Orang Asli* children, our HCPs encountered that there was no way of knowing if the food basket was taken by the particular child. According to the HCPs, food sharing among family members is common. As a result, the child may not benefit optimally from a food basket, as shown by their poor weight gain during mobile clinic visits.

*If a family of five children received just two food baskets with full cream milk powder, the five children would be drinking milk supplies for two (while the other three of these children were not eligible for food aid). As a consequence, it seemed as though the whole family was sharing the two food baskets. They’ll never tell us, but we know since their weights haven’t risen month after month, I visited their home and kitchen. I saw that the milk had been prepared for the whole family rather than a cup for a child (FGD, medical doctor).*

#### Community feeding programs

Supplemental feeding overcomes some of the limitations of food baskets as the food is given to the children at a designated site under the supervision of volunteers. Nevertheless, the challenges associated with this program were that it required the participation of a local village volunteer who could continuously prepare meals for the children five days a week and sustain the service yearly. Additionally, the site must be equipped with food preparation utensils.

*The difference between community feeding and food baskets was that nutrition coverage for children was wider from community feeding than from food baskets…the food basket supplied once a month to the children will be sustained for a month only. But, community feeding is that we feed the children one meal a day for five days a week for every month, and then continuously for a year (IDI, nutritionist).*

## Discussion

The challenges experienced by the HCPs are the inability of *Orang Asli* children to access adequate nutrition and health services, and the need for cross-cultural skills of HCPs to deal with the *Orang Asli* communities and implement a specific nutritional program for them. These findings provide health policymakers a guide to design focused strategies and plans to improve *Orang Asli* children’s care, as well as a different perspective on how to promote indigenous children’s growth in other countries.

Most of the HCPs agreed that poverty was the main contributing factor to making *Orang Asli* children unable to obtain nutritious food. The *Orang Asli* parents, who lack a steady source of income and depend only on forest resources for food, predispose their children to malnutrition. Poverty has been a major problem among indigenous peoples, who account for nearly 15% of the extremely poor worldwide [54]. In this country, approximately 35% of the *Orang Asli* population (0.7% of the Malaysian population) were living below the poverty line income and approximately 20.0% experienced extreme poverty [15, 25, 55]. Poverty was a significant social determinant of malnutrition, with some *Orang Asli* unable to access food. Inadequate purchasing power to get quality food may contribute to the purchase of cheaper energy-dense and nutrient-poor foods to maintain families’ daily dietary intakes at a lower cost [16, 56]. The consumption of these foods may predispose children to growth and development problems due to inadequate nutrients and energy. Besides, the ability to access food among indigenous people has deteriorated as a result of deforestation owing to the loss of natural resources in the forests, which exaggerates the problem of malnutrition among *Orang Asli* children [57].

Several initiatives have been taken by the Ministry of Health in this country to tackle the problem of undernutrition in *Orang Asli* children, such as the introduction of nutrition programs, which may need further improvement. A recent study shows that the success rate of food basket programs is five times lower in *Orang Asli* children than in Malay children [47]. In addition, HCPs lack a structured method to supervise the consumption of food baskets by children. A supplemental feeding that is part of the community feeding program is then in place to further improve the child’s nutritional status [46]. The supplementary feeding program may complement the food basket program. There is also direct supervision via the supplemental feeding program, as children will eat in the community hall under the direct observation of a local village volunteer. The drawback of the supplemental feeding program is that it only provides meals five days a week. Nevertheless, the food basket supply may last a month. A study suggested that nutrition interventions have failed to restore adequate growth rates among impoverished children in many other developing countries [58]. Failures are most often not the consequence of ineffective policies and strategies or a lack of knowledge about suitable remedies, but rather of insufficient program execution and scale. There are several factors believed to contribute to ineffective program implementation globally, including insufficient coordination, a lack of high-level interest, insufficient human resources and capacity, insufficient funding, ineffective strategies, policies with limited sticking power, and structures that impede collaboration [59]. These factors are for policymakers or researchers to consider when evaluating the implementation of the nutrition programme.

Besides their lower socio-economic status, *Orang Asli* communities are geographically isolated from healthcare facilities [15, 56]. Higher expenses are required for *Orang Asli* parents to bring their children to the health facilities, which are located in areas far away from their homes. A study shows that it is a burden for people living in poverty, including indigenous people, to get healthcare services. Underprivileged groups were hindered from seeking healthcare services because of difficult living conditions, poor quality of interaction between providers and underserved patients, and the complexity of healthcare organizations [60]. Despite the availability of mobile clinics for *Orang Asli* that have been established, there are other aspects, including sustainability, budget allocations, and basic amenities and transport, that are factors to be considered when providing mobile health services to the *Orang Asli* who reside in remote areas.

The practice of food taboo prohibits *Orang Asli* children from having a sufficient protein intake, and this increases the difficulty of HCPs in growth management. Despite the *Orang Asli* communities’ food taboos receding as compared to those reported in 1972 [61], some of the taboos persist in their communities. Food taboos have been recognized as a factor in the development of undernutrition in children. According to the UNICEF Food-Care Health conceptual framework, contextual factors, including cultural norms, taboos, and beliefs, are primary factors to be considered for causes of malnutrition [55]. Indigenous people are required to adhere to certain cultural taboos and customs, which affect the food they consume, rendering children more susceptible to protein and micronutrient deficits. Some foods were rejected by the *Orang Asli* because of a kindred spirit or connection with the animal, or because certain animals were considered unclean, or concerns about harmful health consequences associated with the food’s intake [61]. Besides, limited dietary diversity is highlighted as a risk factor for malnutrition, with 58.3% of *Orang Asli* children residing in Selangor state being underweight and 64.3% stunted [22]. Studies found that children who received a lower-protein infant formula had a lower weight and BMI up to the age of six than children who received a higher-protein infant formula [62]. Although diet diversity and high protein consumption are essential for children’s development, our HCPs in this study found it difficult to convince mothers to give their children a diverse diet. Food diversity promotion may be conducted in collaboration with their minor ethnic leader, *Tok Batin*, and *Orang Asli* groups from other tribes who have fewer practiced food taboos [61].

The practice of traditional healing practices that are often linked to spiritual beliefs among *Orang Asli* was another noteworthy information highlighted by the HCPs. This practice hinders HCPs from delivering healthcare services to *Orang Asli* children and preventing them from obtaining care from HCPs. This finding is consistent with the social constructionist approach, which considers how culture and society shape people’s beliefs and understanding of illness, and how this influences their health-seeking behaviour [63]. Their fatalistic beliefs are inextricably linked to indigenous health inequality [64]. The cultural determinants of health impacted indigenous peoples’ ability to receive healthcare services as well as linked with the healthcare service’s ability to comprehend and incorporate indigenous beliefs and values while delivering treatment. Understanding the cultural, historical, and social fabric of the communities by health professionals plays a vital role in making the health services acceptable to the community. Cultural adaptation of health care includes a higher degree of cultural awareness and flexibility in interventions, as well as the participation of community leaders and traditional healers [62]. All HCPs who deal with indigenous people should be aware of these implications for healthcare service delivery.

Cross-cultural skills are essential for HCPs who are involved in managing *Orang Asli* children, who have a distinctly different culture. There is a variety of literature that guides HCPs in caring for indigenous people’s health internationally, addressing cultural, social, and historical aspects of healthcare service accessibility components [30–32, 65]. This guidance highlights the importance of HCPs who are culturally capable as a vital component to improving healthcare delivery and health outcomes for indigenous people with diverse cultures. This includes the abilities of HCPs to work with indigenous belief systems and to engage in culturally appropriate communication [30–32, 65]. The lack of such guidance for the local context has posed a great challenge to HCPs when handling *Orang Asli* children. The challenges identified in this study are important points for health policymakers or researchers to consider if any guidance for HCPs on managing the growth of *Orang Asli* children were to be compiled. This would in turn improve the existing healthcare services for *Orang Asli* children as a whole.

A conceptual framework, "Accessibility Framework for Indigenous peoples accessing Indigenous primary health care services," is the closest framework to enable adaptation for discussing part of the study findings. This framework synthesises the issues associated with indigenous patients, including their families and communities, accessing healthcare services [66]. The framework covers the dimensions of approachability, acceptability, availability, affordability, and ability to engage and is consistent with the context of challenges of accessibility to childhood nutrition and healthcare services among the *Orang Asli* community. Nevertheless, this framework lacks elements that encompass the responsibility of HCPs or the healthcare system in providing healthcare services or access to indigenous people. The element of "access" by the providers is emphasised by Haggerty *et al*., where "access" is defined as the ability of a group of people to attain appropriate healthcare services and should not be limited to the responsibility for access to patients (or recipients) but also providers [67]. Our study incorporates the aspect of providers, as seen in the themes of the skills of healthcare providers and the challenges in the implementation of nutritional programs. Therefore, more research is needed to look at the variety of access from both the supply and demand sides in developing a conceptual framework for indigenous healthcare services.

### Limitations

Our sample population is restricted to Perak state in Malaysia; therefore, the findings may not necessarily be applicable to other states. Nevertheless, the management of growth for *Orang Asli* children is standardized across Peninsular Malaysia. Hence, the findings of this study, although not generalizable, are transferrable to other health settings that provide healthcare services for *Orang Asli* children.

This study’s findings are based on the etic views of HCPs without triangulating with the perspectives of *Orang Asli*. HCPs with as much as 17 years of experience serving these communities are considered qualified to narrate inherent problems in *Orang Asli* communities. Nevertheless, further research on the emic perspectives of the *Orang Asli* population is equally important as their inputs would help health authorities develop culturally competent solutions.

## Conclusion

The challenges of HCPs in managing the growth of *Orang Asli* children are multifactorial, arising from the barriers of the *Orang Asli* community in accessing resources for children’s growth, cultural differences, environmental challenges, and the shortcoming of nutritional programme implementation. This would require a multifaceted approach to address these challenges. This will need the joint efforts of multiple stakeholders, from communities and non-governmental organizations (NGOs) to the Ministry of Health and other policymakers. To bridge the gap between bottom-up initiatives and top-down policies, public health services must be inclusive of and responsive to indigenous peoples’ needs, rather than reinforce the community’s dependence on the state.
